# Crystal structures of anhydrous and hydrated ceftibuten

**DOI:** 10.1107/S2056989022002110

**Published:** 2022-03-10

**Authors:** Matthew L. Nisbet, Marissa Puzan, Lukasz Wojtas, Brian Samas, Geoffrey P. F. Wood

**Affiliations:** a Pharmaceutical Sciences, Pfizer Global Research & Development, Groton, Connecticut 06340, USA; b Department of Chemistry, University of South Florida, 4202 East Fowler Avenue, Tampa, Florida 33620, USA

**Keywords:** crystal structure, ceftibuten, hydrate, hydrogen bonds

## Abstract

Ceftibuten, C_15_H_14_N_4_O_6_S_2_, with the systematic name (6*R*,7*R*)-7-{[(*Z*)-2-(2-amino-1,3-thia­zol-4-yl)-4-carb­oxy­but-2-eno­yl]amino}-8-oxo-5-thia-1-aza­bicyclo­[4.2.0]oct-2-ene-2-carb­oxy­lic acid, is a third generation, orally administered cephalosporin anti­biotic with broad anti­microbial activity and stability against extended spectrum β-lactamases. Ceftibuten can exist in various hydration states and to better understand the location of the water mol­ecules of crystallization and their effect on the structure, the crystal structures of anhydrous (I) and hydrated (II) ceftibuten were determined and both occur as zwitterions with proton transfer from the carboxyl­ate group adjacent to the β-lactam ring to the N atom of the thia­zole ring. The β-lactam ring in (I) is almost planar but the equivalent grouping in (II) is slightly buckled. In the extended structure of (I), O—H⋯O and N—H⋯O hydrogen bonds link the mol­ecules into a three-dimensional network. In (II), O—H⋯O_c_, N—H⋯O_c_, O—H⋯O_w_, N—H⋯O_w_ and O_w_—H⋯O_w_ (c = ceftibuten, w = water) hydrogen bonds link the components into a three-dimensional network. A large void space is present within the anhydrous crystal structure that can accommodate between two and three mol­ecules of water.

## Abstract 2

The crystal structures of anhydrous and hydrated ceftibuten are reported. Both crystallize as zwitterions.

## Chemical context

Ceftibuten, originally marketed under the tradename Cedax in the USA, is a third-generation cephalosporin anti­biotic with activity against a variety of bacterial strains and resistance to extended spectrum β-lactamases (Wiseman & Balfour, 1994 ▸; Hamashima *et al.*, 1990 ▸). Oral administration of ceftibuten is effective for treating urinary tract or respiratory tract infections, including many caused by β-lactamase-expressing bacterial strains (Owens *et al.*, 1997 ▸). Despite its withdrawal from the US market, because of its effectiveness and stability against β-lactamases, renewed inter­est in ceftibuten for multi-drug-resistant urinary tract infections (UTIs) has emerged, and studies are underway investigating oral administration of ceftibuten co-administered with a β-lactamase inhibitor as an alternative to hospitalization for complicated UTIs (Veeraraghavan *et al.*, 2021 ▸; Chatwin *et al.*, 2021 ▸).

Despite its long-time commercial availability, to our knowledge no crystal structures of ceftibuten have been previously reported. The structures of anhydrous ceftibuten (I) and hydrated ceftibuten (II) are reported herein. 
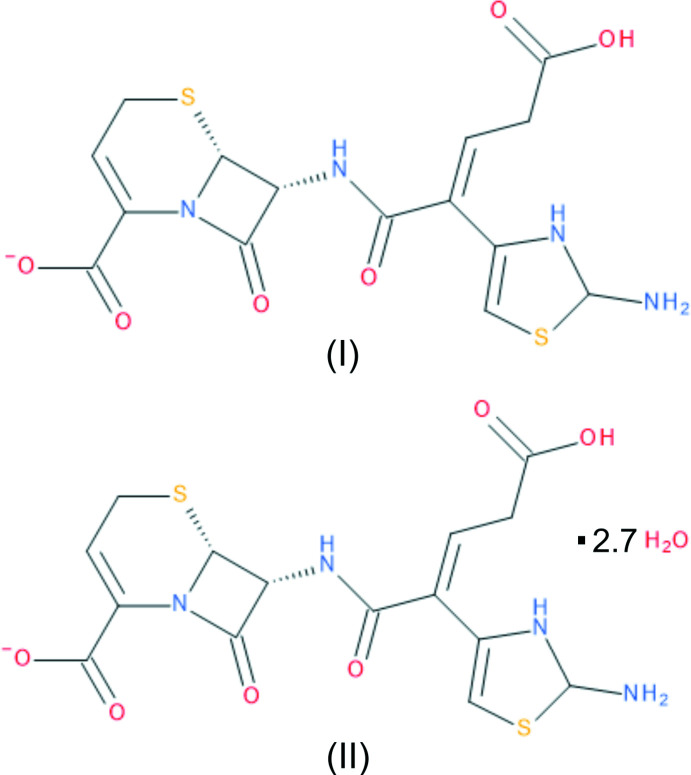


## Structural commentary

**Figure 1:**
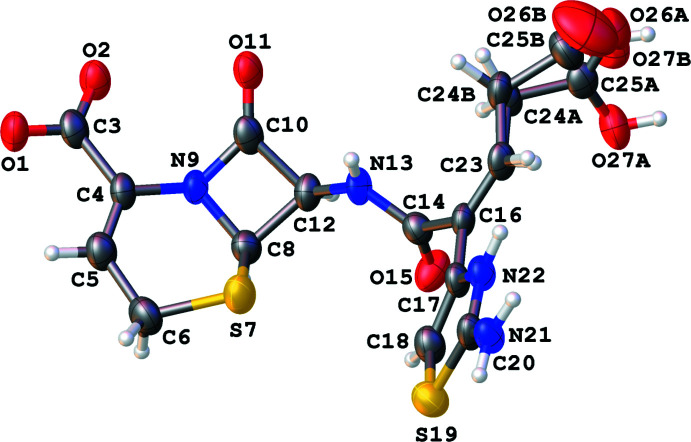
Mol­ecular structure of (I). Ellipsoids of non-H elements are drawn at 50% probability.

The anhydrous compound (I) (Fig. 1 ▸) has the formula C_15_H_14_N_4_O_6_S_2_ and crystallizes in the ortho­rhom­bic space group *P*2_1_2_1_2_1_. The asymmetric unit of (I) contains one mol­ecule of ceftibuten: the chiral C8 and C12 centers both have an absolute configuration of *R*. This is reflected in the N13—C12—C8—S7 torsion angle of 5.0 (10)°. The C24—C25—O26—O27 atoms were treated as disordered over two adjacent sets of sites with a population ratio of 0.841 (11): 0.159 (11). The β-lactam ring is almost planar with the C8/C12/C10/N9 atoms in the ring having a calculated r.m.s. deviation of 0.032 Å. Based on the refined bond distances of C3—O1 = 1.258 (9) Å and C3—O2 = 1.254 (9) Å, we have assigned the O1—O2—C3 group as a carboxyl­ate and the N22 atom of the thia­zole ring as protonated based on peaks in the residual electron density map, *i.e*., the mol­ecule exists as a zwitterion in the solid state.

**Figure 2:**
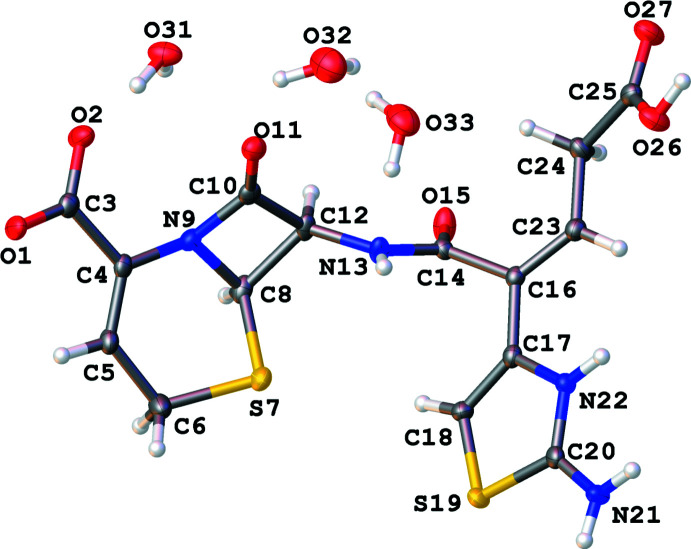
Mol­ecular structure of (II). Ellipsoids of non-H elements are drawn at 50% probability.

The hydrated compound (II) (Fig. 2 ▸) has the formula C_15_H_14_N_4_O_6_S_2_·2.7H_2_O and crystallizes in the ortho­rhom­bic space group *P*2_1_2_1_2_1_ with similar unit-cell parameters to (I). The asymmetric unit of (II) includes one ceftibuten mol­ecule, one fully occupied O31 water mol­ecule, and two partially occupied O32 and O33 water mol­ecules, which were independently refined to occupancies of 0.828 (10) and 0.824 (12), respectively. The chiral C8 and C12 centers both have an absolute configuration of *R* and N13—C12—C8—S7 = 17.2 (4)°. The β-lactam ring is slightly buckled in (II) compared to (I), with the atoms in the ring having a calculated r.m.s. deviation of 0.078 Å. As in (I), we have assigned the O1—C3—O2 group as a carboxyl­ate anion based on bond distances of C3—O1 = 1.252 (4) Å and C3—O2 = 1.256 (4) Å and the N22 atom as protonated based on peaks in the residual electron-density map.

## Supra­molecular features

**Figure 3:**
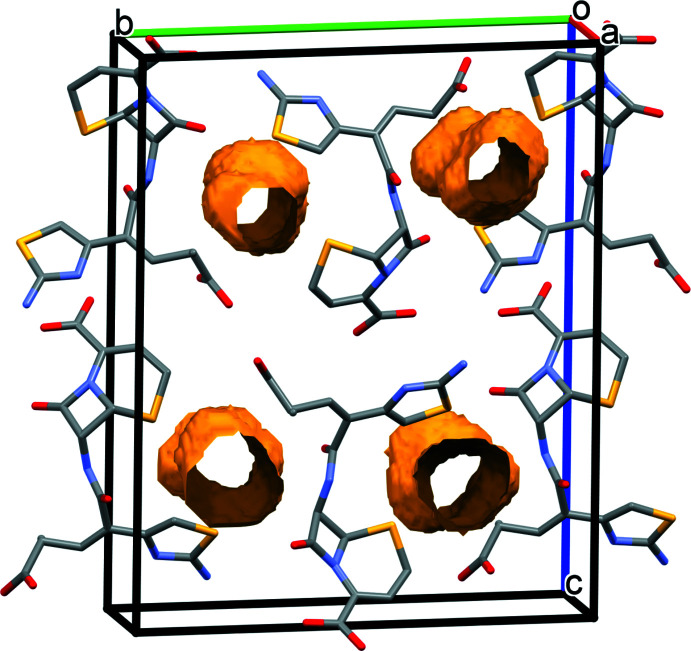
Packing diagram of (I). Void spaces are shown in orange. Hydrogen atoms are omitted for clarity.

The extended structure of (I) displays a three-dimensional hydrogen-bonding network with O—H⋯O and N—H⋯O hydrogen bonds linking adjacent ceftibuten mol­ecules (Table 1 ▸). The structure of (I) contains four void spaces per unit cell of about 42 Å^3^ each (total void volume = 167.3 Å^3^), which account for 9.2% of the unit-cell volume, as calculated in *PLATON* (Spek, 2020 ▸). The void spaces form channels propagating along the [100] direction (Fig. 3 ▸). The layers of ceftibuten mol­ecules are linked along the *a*-axis direction by N—H⋯O hydrogen bonds. Two weak C—H⋯O inter­actions are also present.

**Figure 4:**
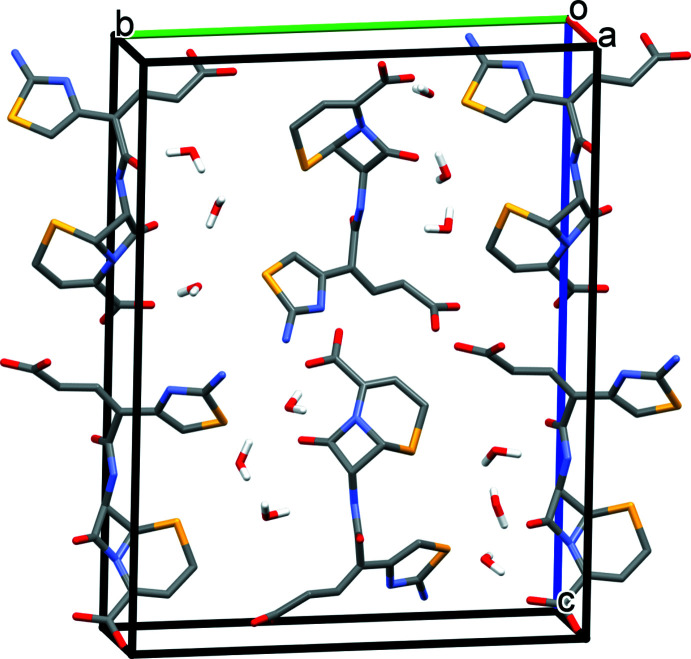
Packing diagram of (II). Non-water H atoms are omitted for clarity.

Compound (II) displays a three-dimensional hydrogen-bonding network composed of O—H⋯O and N—H⋯O hydrogen bonds between ceftibuten mol­ecules, O—H⋯O and N—H⋯O hydrogen bonds between ceftibuten and the free water mol­ecules, and O—H⋯O hydrogen bonds between the free water mol­ecules (Table 2 ▸). Four weak C—H⋯O bonds occur. The O32 and O33 water mol­ecules occupy the channel void space that is present in (I) (Fig. 4 ▸).

## Database survey

A Cambridge Structural Database search for compounds containing a β-lactam ring resulted in 1381 hits [CSD version 5.42 (December 2020), ConQuest version 2020.3.0; Groom *et al.*, 2016 ▸]. Atoms in the β-lactam rings in these compounds have an average r.m.s. deviation of 0.024 Å, with the r.m.s. deviations of atoms in the β-lactam rings in (I) and (II) falling in the 69th and 98th percentiles of the distribution, respectively.

A previous study examined the structures of 32 known water-containing β-lactams (Hickey *et al.*, 2007 ▸). Following the system of Gillon *et al.* (2003 ▸), the authors describe three distinct hydrogen-bonding motifs in hydrated β-lactam compounds based on the donor/acceptor roles of the water mol­ecules in hydrogen bonds. The O31 water mol­ecule in (II) acts as a donor in two hydrogen bonds and acceptor in two hydrogen bonds, meaning that the hydrogen-bonding behavior of the O31 water mol­ecule in (II) can be classified as ‘environment C′. In contrast, the O32 and O33 water mol­ecules can be assigned environment B based on their participation as donors in two hydrogen bonds and as acceptors in one hydrogen bond.

## Synthesis and crystallization

Ceftibuten hydrate was purchased from ACS Dobfar (Tribiano, Italy). Dehydration occurs following exposure to an atmosphere below 30% relative humidity at 298 K, and the material was confirmed to be anhydrous following receipt at the University of South Florida X-Ray Facility. A crystal in the form of a colorless needle was selected directly from the bulk sample (I) and deemed suitable for analysis.

For rehydration, ceftibuten powder was placed in an uncapped scintillation vial within a container of pure water. The sealed container was stored at room temperature for four weeks, and a sufficiently large crystal (a colorless needle) was selected for analysis.

## Refinement

Crystal data, data collection and structure refinement details are summarized in Table 3 ▸. The N—H and O—H hydrogen positions were assigned from residual electron density peaks and refined with distances constrained. All remaining hydrogen atoms were assigned with a riding model. The C24—C25—O26—O27 atoms in (I) were treated as disordered with a population ratio of approximately 80:20 and refined with restrained inter­atomic distances. The occupancies of the O32 and O33 water mol­ecules in (II) were freely refined.

## Supplementary Material

Crystal structure: contains datablock(s) I, II. DOI: 10.1107/S2056989022002110/hb8008sup1.cif

Structure factors: contains datablock(s) I. DOI: 10.1107/S2056989022002110/hb8008Isup3.hkl

Structure factors: contains datablock(s) II. DOI: 10.1107/S2056989022002110/hb8008IIsup2.hkl

Supporting information file. DOI: 10.1107/S2056989022002110/hb8008Isup4.cml

Supporting information file. DOI: 10.1107/S2056989022002110/hb8008IIsup5.cml

CCDC references: 2154016, 2154015

Additional supporting information:  crystallographic information; 3D view; checkCIF report

## Figures and Tables

**Table 1:** Hydrogen-bond geometry (Å, °) for (I)

*D*—H⋯*A*	*D*—H	H⋯*A*	*D*⋯*A*	*D*—H⋯*A*
N13—H13⋯O15^i^	0.90 (3)	1.91 (3)	2.807 (9)	177 (7)
N21—H21*A*⋯O2^ii^	0.87 (3)	2.02 (5)	2.824 (8)	153 (8)
N21—H21*B*⋯O2^iii^	0.88 (3)	1.96 (4)	2.816 (9)	164 (8)
N22—H22⋯O1^iii^	0.89 (3)	1.75 (3)	2.637 (9)	172 (9)
O27*A*—H27*A*⋯O26*A* ^iv^	0.84	1.85	2.683 (9)	170
O27*B*—H27*B*⋯O26*B* ^iv^	0.84	1.84	2.62 (6)	154
C12—H12⋯O11^v^	1.00	2.27	3.172 (10)	150
C23—H23⋯O1^iii^	0.95	2.35	3.237 (9)	156

Symmetry codes: (i) 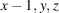 ; (ii) 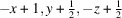 ; (iii) 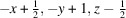 ; (iv) 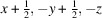 ; (v) 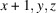 .

**Table 2:** Hydrogen-bond geometry (Å, °) for (II)

*D*—H⋯*A*	*D*—H	H⋯*A*	*D*⋯*A*	*D*—H⋯*A*
N13—H13⋯O15^i^	0.85 (2)	2.01 (3)	2.799 (4)	154 (4)
N21—H21*A*⋯O31^ii^	0.86 (5)	2.05 (5)	2.838 (4)	153 (4)
N21—H21*B*⋯O2^iii^	0.85 (4)	1.97 (5)	2.811 (4)	173 (4)
N22—H22⋯O1^iii^	0.87 (5)	1.78 (5)	2.654 (4)	178 (5)
O26—H26⋯O27^iv^	0.87 (5)	1.80 (5)	2.647 (4)	164 (4)
O31—H31*A*⋯O2^v^	0.85 (2)	2.29 (3)	3.071 (4)	154 (5)
O31—H31*B*⋯O2	0.86 (3)	1.91 (3)	2.756 (4)	167 (6)
O32—H32*A*⋯O33^v^	0.88 (3)	2.02 (3)	2.874 (6)	164 (8)
O32—H32*B*⋯O31^i^	0.87 (3)	2.46 (3)	3.305 (5)	167 (6)
O33—H33*A*⋯O15^vi^	0.87 (8)	2.40 (8)	3.226 (5)	159 (6)
O33—H33*B*⋯O32	0.88 (8)	1.97 (8)	2.837 (6)	165 (6)
C12—H12⋯O11^v^	1.00	2.39	3.349 (4)	161
C23—H23⋯O1^iii^	0.95	2.41	3.281 (4)	152
C24—H24*B*⋯O26^v^	0.99	2.54	3.387 (5)	143

Symmetry codes: (i) 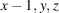 ; (ii) 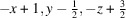 ; (iii) 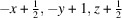 ; (iv) 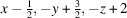 ; (v) 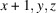 ; (vi) 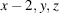 .

**Table 3:** Experimental details

	(I)	(II)
Crystal data		
Chemical formula	C_15_H_14_N_4_O_6_S_2_	C_15_H_14_N_4_O_6_S_2_·2.652H_2_O
*M*_r_	410.42	458.21
Crystal system, space group	Orthorhombic, *P*2_1_2_1_2_1_	Orthorhombic, *P*2_1_2_1_2_1_
Temperature (K)	100	100
*a*, *b*, *c* (Å)	4.7727 (2), 17.5228 (8), 21.8526 (9)	4.6690 (1), 17.8029 (4), 23.1486 (5)
*V* (Å^3^)	1827.56 (14)	1924.15 (7)
*Z*	4	4
Radiation type	Cu *K*α	Cu *K*α
μ (mm^−1^)	3.02	3.04
Crystal size (mm)	0.04 × 0.01 × 0.01	0.1 × 0.02 × 0.02

Data collection		
Diffractometer	Bruker D8 Venture Photon-II CPAD	Bruker D8 Venture Photon-II CPAD
Absorption correction	Multi-scan (*SADABS*; Bruker, 2016 ▸)	Multi-scan (*SADABS*; Bruker, 2016 ▸)
*T*_min_, *T*_max_	0.790, 1.000	0.919, 1.000
No. of measured, independent and observed [*I* > 2σ(*I*)] reflections	14411, 3215, 1954	26013, 4040, 3605
*R*_int_	0.128	0.070
(sin θ/λ)_max_ (Å^−1^)	0.603	0.634

Refinement		
*R*[*F* ^2^ > 2σ(*F* ^2^)], *wR*(*F* ^2^), *S*	0.060, 0.146, 1.02	0.038, 0.085, 1.04
No. of reflections	3215	4040
No. of parameters	297	317
No. of restraints	127	7
H-atom treatment	H atoms treated by a mixture of independent and constrained refinement	H atoms treated by a mixture of independent and constrained refinement
Δρ_max_, Δρ_min_ (e Å^−3^)	0.25, −0.31	0.30, −0.23
Absolute structure	Flack *x* determined using 544 quotients [(*I* ^+^)−(*I* ^−^)]/[(*I* ^+^)+(*I* ^−^)] (Parsons *et al.*, 2013 ▸)	Flack *x* determined using 1302 quotients [(*I* ^+^)−(*I* ^−^)]/[(*I* ^+^)+(*I* ^−^)] (Parsons *et al.*, 2013 ▸)
Absolute structure parameter	0.02 (3)	0.029 (11)

Computer programs: *SAINT* (Bruker, 2016 ▸), *SHELXT* (Sheldrick, 2015*a* ▸), *SHELXL* (Sheldrick, 2015*b* ▸), and *OLEX2* (Dolomanov *et al.*, 2009 ▸).

## supplementary crystallographic information

### (6R,7R)-7-{[(Z)-2-(2-Amino-1,3-thiazol-4-yl)-4-carboxybut-2-enoyl]amino}-8-oxo-5-thia-1-azabicyclo[4.2.0]oct-2-ene-2-carboxylic acid (I) Crystal data

**Table:**

C_15_H_14_N_4_O_6_S_2_	*D*_x_ = 1.492 Mg m^−^^3^
*M**_r_* = 410.42	Cu *K*α radiation, λ = 1.54178 Å
Orthorhombic, *P*2_1_2_1_2_1_	Cell parameters from 3229 reflections
*a* = 4.7727 (2) Å	θ = 3.2–66.0°
*b* = 17.5228 (8) Å	µ = 3.02 mm^−^^1^
*c* = 21.8526 (9) Å	*T* = 100 K
*V* = 1827.56 (14) Å^3^	Needle, colourless
*Z* = 4	0.04 × 0.01 × 0.01 mm
*F*(000) = 848	

### (6R,7R)-7-{[(Z)-2-(2-Amino-1,3-thiazol-4-yl)-4-carboxybut-2-enoyl]amino}-8-oxo-5-thia-1-azabicyclo[4.2.0]oct-2-ene-2-carboxylic acid (I) Data collection

**Table:**

Bruker D8 Venture Photon-II CPAD diffractometer	1954 reflections with *I* > 2σ(*I*)
ω scans	*R*_int_ = 0.128
Absorption correction: multi-scan (SADABS; Bruker, 2016)	θ_max_ = 68.3°, θ_min_ = 3.2°
*T*_min_ = 0.790, *T*_max_ = 1.000	*h* = −5→5
14411 measured reflections	*k* = −20→20
3215 independent reflections	*l* = −25→25

### (6R,7R)-7-{[(Z)-2-(2-Amino-1,3-thiazol-4-yl)-4-carboxybut-2-enoyl]amino}-8-oxo-5-thia-1-azabicyclo[4.2.0]oct-2-ene-2-carboxylic acid (I) Refinement

**Table:**

Refinement on *F*^2^	Hydrogen site location: mixed
Least-squares matrix: full	H atoms treated by a mixture of independent and constrained refinement
*R*[*F*^2^ > 2σ(*F*^2^)] = 0.060	*w* = 1/[σ^2^(*F*_o_^2^) + (0.0594*P*)^2^ + 0.1157*P*] where *P* = (*F*_o_^2^ + 2*F*_c_^2^)/3
*wR*(*F*^2^) = 0.146	(Δ/σ)_max_ < 0.001
*S* = 1.02	Δρ_max_ = 0.25 e Å^−^^3^
3215 reflections	Δρ_min_ = −0.30 e Å^−^^3^
297 parameters	Absolute structure: Flack *x* determined using 544 quotients [(*I*^+^)-(*I*^-^)]/[(*I*^+^)+(*I*^-^)] (Parsons *et al.*, 2013)
127 restraints	Absolute structure parameter: 0.02 (3)

### (6R,7R)-7-{[(Z)-2-(2-Amino-1,3-thiazol-4-yl)-4-carboxybut-2-enoyl]amino}-8-oxo-5-thia-1-azabicyclo[4.2.0]oct-2-ene-2-carboxylic acid (I) Special details

**Table:**

Geometry. All esds (except the esd in the dihedral angle between two l.s. planes) are estimated using the full covariance matrix. The cell esds are taken into account individually in the estimation of esds in distances, angles and torsion angles; correlations between esds in cell parameters are only used when they are defined by crystal symmetry. An approximate (isotropic) treatment of cell esds is used for estimating esds involving l.s. planes.

### (6R,7R)-7-{[(Z)-2-(2-Amino-1,3-thiazol-4-yl)-4-carboxybut-2-enoyl]amino}-8-oxo-5-thia-1-azabicyclo[4.2.0]oct-2-ene-2-carboxylic acid (I) Fractional atomic coordinates and isotropic or equivalent isotropic displacement parameters (Å^2^)

**Table:**

	*x*	*y*	*z*	*U*_iso_*/*U*_eq_	Occ. (<1)
O1	0.1910 (13)	0.4734 (3)	0.5240 (2)	0.0496 (15)	
O2	0.3613 (13)	0.3637 (3)	0.4870 (2)	0.0478 (15)	
C3	0.3498 (19)	0.4351 (5)	0.4898 (3)	0.046 (2)	
C4	0.5398 (18)	0.4812 (4)	0.4497 (3)	0.0386 (19)	
C5	0.577 (2)	0.5565 (4)	0.4568 (3)	0.048 (2)	
H5	0.477736	0.579712	0.489583	0.057*	
C6	0.756 (2)	0.6079 (5)	0.4189 (3)	0.053 (2)	
H6A	0.943643	0.611709	0.437976	0.064*	
H6B	0.672455	0.659595	0.418555	0.064*	
S7	0.7947 (5)	0.57482 (13)	0.34071 (8)	0.0534 (6)	
C8	0.9042 (19)	0.4805 (4)	0.3648 (3)	0.043 (2)	
H8	1.093338	0.480121	0.384487	0.052*	
N9	0.6880 (14)	0.4440 (3)	0.4024 (2)	0.0381 (16)	
C10	0.626 (2)	0.3886 (4)	0.3593 (3)	0.043 (2)	
O11	0.4465 (13)	0.3410 (3)	0.3547 (2)	0.0452 (14)	
C12	0.8659 (17)	0.4161 (4)	0.3178 (3)	0.041 (2)	
H12	1.027651	0.379824	0.317186	0.049*	
N13	0.7684 (15)	0.4356 (4)	0.2567 (2)	0.0389 (16)	
H13	0.587 (7)	0.442 (4)	0.247 (3)	0.047*	
C14	0.955 (2)	0.4546 (4)	0.2131 (3)	0.0386 (19)	
O15	1.2075 (14)	0.4538 (3)	0.2209 (2)	0.0522 (16)	
C16	0.8241 (17)	0.4785 (4)	0.1533 (3)	0.0360 (18)	
C17	0.8370 (17)	0.5608 (4)	0.1416 (3)	0.0373 (19)	
C18	1.0011 (19)	0.6113 (4)	0.1705 (3)	0.047 (2)	
H18	1.132680	0.598209	0.201402	0.057*	
S19	0.9437 (5)	0.70346 (12)	0.14471 (9)	0.0518 (6)	
C20	0.6983 (19)	0.6708 (4)	0.0931 (3)	0.045 (2)	
N21	0.5589 (17)	0.7123 (4)	0.0538 (3)	0.0439 (17)	
H21A	0.586 (19)	0.7617 (17)	0.054 (4)	0.06 (3)*	
H21B	0.448 (15)	0.690 (4)	0.027 (3)	0.07 (3)*	
N22	0.6717 (15)	0.5942 (4)	0.0971 (2)	0.0402 (17)	
H22	0.548 (16)	0.568 (4)	0.075 (4)	0.09 (4)*	
C23	0.6960 (18)	0.4289 (4)	0.1170 (3)	0.0421 (19)	
H23	0.609951	0.447977	0.080944	0.051*	0.841 (11)
H23A	0.641091	0.447275	0.077935	0.051*	0.159 (11)
C24A	0.677 (4)	0.3432 (5)	0.1291 (4)	0.044 (4)	0.841 (11)
H24A	0.792881	0.329912	0.165094	0.053*	0.841 (11)
H24B	0.480167	0.329273	0.138372	0.053*	0.841 (11)
C25A	0.773 (3)	0.3002 (6)	0.0761 (4)	0.042 (3)	0.841 (11)
O26A	0.6199 (19)	0.2666 (5)	0.0399 (3)	0.064 (3)	0.841 (11)
O27A	1.0463 (18)	0.3017 (4)	0.0688 (3)	0.058 (2)	0.841 (11)
H27A	1.087486	0.282934	0.034547	0.087*	0.841 (11)
C24B	0.63 (2)	0.3455 (15)	0.1308 (17)	0.050 (15)	0.159 (11)
H24C	0.781827	0.322157	0.154177	0.060*	0.159 (11)
H24D	0.454938	0.342949	0.156221	0.060*	0.159 (11)
C25B	0.582 (10)	0.3031 (19)	0.0741 (18)	0.063 (12)	0.159 (11)
O26B	0.401 (12)	0.318 (3)	0.037 (2)	0.130 (19)	0.159 (11)
O27B	0.760 (13)	0.247 (3)	0.066 (3)	0.118 (19)	0.159 (11)
H27B	0.777928	0.237818	0.028830	0.177*	0.159 (11)

### (6R,7R)-7-{[(Z)-2-(2-Amino-1,3-thiazol-4-yl)-4-carboxybut-2-enoyl]amino}-8-oxo-5-thia-1-azabicyclo[4.2.0]oct-2-ene-2-carboxylic acid (I) Atomic displacement parameters (Å^2^)

**Table:**

	*U*^11^	*U*^22^	*U*^33^	*U*^12^	*U*^13^	*U*^23^
O1	0.066 (4)	0.048 (3)	0.035 (3)	0.004 (3)	0.013 (3)	−0.003 (2)
O2	0.068 (4)	0.042 (3)	0.034 (3)	0.006 (3)	0.004 (3)	0.002 (2)
C3	0.059 (6)	0.054 (6)	0.023 (4)	0.003 (5)	−0.006 (4)	−0.002 (4)
C4	0.048 (6)	0.047 (5)	0.021 (3)	0.004 (4)	−0.001 (4)	−0.002 (3)
C5	0.062 (6)	0.050 (5)	0.031 (4)	0.001 (5)	−0.001 (4)	−0.001 (4)
C6	0.067 (7)	0.046 (5)	0.046 (4)	0.001 (5)	0.002 (5)	−0.011 (4)
S7	0.0756 (17)	0.0481 (12)	0.0365 (10)	−0.0027 (12)	0.0074 (11)	0.0017 (9)
C8	0.054 (6)	0.046 (5)	0.029 (4)	−0.005 (4)	−0.001 (4)	−0.001 (3)
N9	0.050 (4)	0.042 (4)	0.022 (3)	−0.002 (3)	0.005 (3)	−0.001 (2)
C10	0.063 (7)	0.040 (5)	0.025 (4)	0.008 (5)	−0.004 (4)	0.004 (3)
O11	0.065 (4)	0.046 (3)	0.025 (2)	0.002 (3)	0.000 (3)	0.002 (2)
C12	0.048 (6)	0.050 (5)	0.025 (4)	0.005 (4)	0.002 (4)	0.001 (3)
N13	0.050 (5)	0.050 (4)	0.017 (3)	0.003 (4)	−0.003 (3)	0.002 (3)
C14	0.046 (6)	0.039 (4)	0.031 (4)	−0.001 (4)	−0.004 (4)	−0.003 (3)
O15	0.040 (4)	0.080 (4)	0.037 (3)	0.003 (3)	−0.002 (3)	0.013 (3)
C16	0.044 (5)	0.044 (4)	0.020 (3)	0.000 (4)	0.005 (3)	−0.001 (3)
C17	0.051 (5)	0.042 (5)	0.019 (3)	0.001 (4)	0.001 (4)	−0.001 (3)
C18	0.063 (7)	0.046 (5)	0.033 (4)	0.002 (4)	0.000 (4)	−0.002 (3)
S19	0.0628 (15)	0.0459 (12)	0.0466 (11)	−0.0040 (12)	−0.0053 (11)	−0.0022 (10)
C20	0.069 (7)	0.041 (5)	0.025 (4)	−0.002 (5)	0.008 (4)	0.000 (3)
N21	0.068 (5)	0.035 (4)	0.029 (3)	−0.002 (4)	0.000 (4)	0.002 (3)
N22	0.051 (5)	0.044 (5)	0.025 (3)	−0.001 (4)	0.000 (3)	−0.001 (3)
C23	0.060 (5)	0.045 (5)	0.021 (3)	0.004 (4)	0.001 (4)	0.006 (3)
C24A	0.063 (10)	0.041 (6)	0.029 (6)	0.001 (6)	−0.003 (6)	−0.003 (5)
C25A	0.059 (8)	0.037 (6)	0.029 (5)	0.003 (6)	−0.002 (5)	0.008 (4)
O26A	0.074 (7)	0.083 (6)	0.034 (4)	−0.013 (5)	−0.004 (4)	−0.018 (4)
O27A	0.072 (6)	0.065 (5)	0.036 (4)	0.010 (5)	−0.001 (4)	−0.013 (3)
C24B	0.07 (3)	0.05 (3)	0.03 (2)	0.00 (2)	0.01 (2)	0.00 (2)
C25B	0.09 (2)	0.06 (2)	0.043 (19)	−0.01 (2)	−0.001 (18)	−0.015 (17)
O26B	0.15 (3)	0.12 (4)	0.11 (3)	−0.01 (3)	−0.07 (3)	−0.02 (3)
O27B	0.14 (4)	0.09 (3)	0.13 (4)	0.02 (3)	0.01 (3)	−0.05 (3)

### (6R,7R)-7-{[(Z)-2-(2-Amino-1,3-thiazol-4-yl)-4-carboxybut-2-enoyl]amino}-8-oxo-5-thia-1-azabicyclo[4.2.0]oct-2-ene-2-carboxylic acid (I) Geometric parameters (Å, º)

**Table:**

O1—C3	1.258 (9)	C17—N22	1.382 (9)
O2—C3	1.254 (9)	C18—H18	0.9500
C3—C4	1.497 (11)	C18—S19	1.732 (8)
C4—C5	1.340 (10)	S19—C20	1.724 (8)
C4—N9	1.412 (9)	C20—N21	1.307 (10)
C5—H5	0.9500	C20—N22	1.351 (10)
C5—C6	1.493 (11)	N21—H21A	0.87 (3)
C6—H6A	0.9900	N21—H21B	0.88 (3)
C6—H6B	0.9900	N22—H22	0.89 (3)
C6—S7	1.814 (8)	C23—H23	0.9500
S7—C8	1.812 (8)	C23—H23A	0.9500
C8—H8	1.0000	C23—C24A	1.527 (11)
C8—N9	1.467 (10)	C23—C24B	1.527 (16)
C8—C12	1.536 (10)	C24A—H24A	0.9900
N9—C10	1.384 (9)	C24A—H24B	0.9900
C10—O11	1.202 (9)	C24A—C25A	1.456 (14)
C10—C12	1.536 (11)	C25A—O26A	1.226 (12)
C12—H12	1.0000	C25A—O27A	1.315 (12)
C12—N13	1.455 (8)	O27A—H27A	0.8400
N13—H13	0.90 (3)	C24B—H24C	0.9900
N13—C14	1.345 (10)	C24B—H24D	0.9900
C14—O15	1.220 (9)	C24B—C25B	1.460 (18)
C14—C16	1.506 (10)	C25B—O26B	1.21 (2)
C16—C17	1.466 (10)	C25B—O27B	1.31 (2)
C16—C23	1.327 (10)	O27B—H27B	0.8400
C17—C18	1.340 (10)		
			
O1—C3—C4	115.1 (7)	C18—C17—C16	126.3 (7)
O2—C3—O1	126.0 (8)	C18—C17—N22	112.7 (7)
O2—C3—C4	118.9 (7)	N22—C17—C16	121.0 (7)
C5—C4—C3	122.9 (7)	C17—C18—H18	124.2
C5—C4—N9	118.2 (7)	C17—C18—S19	111.7 (6)
N9—C4—C3	118.9 (6)	S19—C18—H18	124.2
C4—C5—H5	116.4	C20—S19—C18	90.6 (4)
C4—C5—C6	127.3 (7)	N21—C20—S19	126.2 (7)
C6—C5—H5	116.4	N21—C20—N22	123.2 (8)
C5—C6—H6A	109.0	N22—C20—S19	110.5 (6)
C5—C6—H6B	109.0	C20—N21—H21A	118 (6)
C5—C6—S7	112.8 (5)	C20—N21—H21B	120 (6)
H6A—C6—H6B	107.8	H21A—N21—H21B	122 (8)
S7—C6—H6A	109.0	C17—N22—H22	123 (6)
S7—C6—H6B	109.0	C20—N22—C17	114.4 (7)
C8—S7—C6	92.7 (3)	C20—N22—H22	123 (6)
S7—C8—H8	113.0	C16—C23—H23	117.6
N9—C8—S7	111.0 (6)	C16—C23—H23A	116.2
N9—C8—H8	113.0	C16—C23—C24A	124.7 (8)
N9—C8—C12	88.3 (5)	C16—C23—C24B	128 (2)
C12—C8—S7	116.2 (5)	C24A—C23—H23	117.6
C12—C8—H8	113.0	C24B—C23—H23A	116.2
C4—N9—C8	124.2 (6)	C23—C24A—H24A	109.5
C10—N9—C4	135.7 (7)	C23—C24A—H24B	109.5
C10—N9—C8	94.2 (5)	H24A—C24A—H24B	108.1
N9—C10—C12	91.4 (6)	C25A—C24A—C23	110.6 (8)
O11—C10—N9	134.1 (7)	C25A—C24A—H24A	109.5
O11—C10—C12	134.4 (7)	C25A—C24A—H24B	109.5
C8—C12—H12	112.6	O26A—C25A—C24A	125.0 (14)
C10—C12—C8	85.7 (5)	O26A—C25A—O27A	121.4 (9)
C10—C12—H12	112.6	O27A—C25A—C24A	113.6 (12)
N13—C12—C8	118.6 (6)	C25A—O27A—H27A	109.5
N13—C12—C10	112.2 (7)	C23—C24B—H24C	109.5
N13—C12—H12	112.6	C23—C24B—H24D	109.5
C12—N13—H13	124 (5)	H24C—C24B—H24D	108.1
C14—N13—C12	119.9 (7)	C25B—C24B—C23	111 (2)
C14—N13—H13	116 (5)	C25B—C24B—H24C	109.5
N13—C14—C16	114.2 (8)	C25B—C24B—H24D	109.5
O15—C14—N13	123.5 (7)	O26B—C25B—C24B	124 (4)
O15—C14—C16	122.3 (7)	O26B—C25B—O27B	123 (4)
C17—C16—C14	114.1 (6)	O27B—C25B—C24B	114 (4)
C23—C16—C14	121.7 (7)	C25B—O27B—H27B	109.5
C23—C16—C17	124.1 (6)		
			
O1—C3—C4—C5	11.5 (11)	C12—C8—N9—C10	−5.2 (6)
O1—C3—C4—N9	−168.8 (7)	C12—N13—C14—O15	2.8 (11)
O2—C3—C4—C5	−168.4 (8)	C12—N13—C14—C16	−176.1 (6)
O2—C3—C4—N9	11.4 (11)	N13—C14—C16—C17	105.8 (8)
C3—C4—C5—C6	−178.8 (8)	N13—C14—C16—C23	−70.5 (10)
C3—C4—N9—C8	−172.5 (7)	C14—C16—C17—C18	15.9 (11)
C3—C4—N9—C10	42.2 (11)	C14—C16—C17—N22	−164.4 (7)
C4—C5—C6—S7	28.5 (12)	C14—C16—C23—C24A	−3.0 (15)
C4—N9—C10—O11	−19.3 (14)	C14—C16—C23—C24B	8 (5)
C4—N9—C10—C12	157.0 (8)	O15—C14—C16—C17	−73.1 (9)
C5—C4—N9—C8	7.2 (11)	O15—C14—C16—C23	110.6 (10)
C5—C4—N9—C10	−138.0 (8)	C16—C17—C18—S19	−178.0 (6)
C5—C6—S7—C8	−52.2 (7)	C16—C17—N22—C20	177.7 (7)
C6—S7—C8—N9	59.2 (6)	C16—C23—C24A—C25A	−127.6 (12)
C6—S7—C8—C12	158.0 (7)	C16—C23—C24B—C25B	−159 (3)
S7—C8—N9—C4	−44.1 (8)	C17—C16—C23—C24A	−179.0 (11)
S7—C8—N9—C10	112.3 (5)	C17—C16—C23—C24B	−168 (5)
S7—C8—C12—C10	−107.9 (6)	C17—C18—S19—C20	−1.1 (6)
S7—C8—C12—N13	5.0 (10)	C18—C17—N22—C20	−2.5 (9)
C8—N9—C10—O11	−171.1 (9)	C18—S19—C20—N21	−178.7 (8)
C8—N9—C10—C12	5.2 (6)	C18—S19—C20—N22	−0.3 (6)
C8—C12—N13—C14	88.2 (9)	S19—C20—N22—C17	1.6 (8)
N9—C4—C5—C6	1.4 (13)	N21—C20—N22—C17	−179.9 (7)
N9—C8—C12—C10	4.7 (5)	N22—C17—C18—S19	2.2 (9)
N9—C8—C12—N13	117.6 (7)	C23—C16—C17—C18	−167.9 (8)
N9—C10—C12—C8	−4.9 (6)	C23—C16—C17—N22	11.9 (12)
N9—C10—C12—N13	−124.1 (6)	C23—C24A—C25A—O26A	−104.7 (14)
C10—C12—N13—C14	−174.4 (6)	C23—C24A—C25A—O27A	73.7 (15)
O11—C10—C12—C8	171.3 (9)	C23—C24B—C25B—O26B	−61 (7)
O11—C10—C12—N13	52.2 (12)	C23—C24B—C25B—O27B	119 (7)
C12—C8—N9—C4	−161.7 (7)		

### (6R,7R)-7-{[(Z)-2-(2-Amino-1,3-thiazol-4-yl)-4-carboxybut-2-enoyl]amino}-8-oxo-5-thia-1-azabicyclo[4.2.0]oct-2-ene-2-carboxylic acid (I) Hydrogen-bond geometry (Å, º)

**Table:**

*D*—H···*A*	*D*—H	H···*A*	*D*···*A*	*D*—H···*A*
N13—H13···O15^i^	0.90 (3)	1.91 (3)	2.807 (9)	177 (7)
N21—H21*A*···O2^ii^	0.87 (3)	2.02 (5)	2.824 (8)	153 (8)
N21—H21*B*···O2^iii^	0.88 (3)	1.96 (4)	2.816 (9)	164 (8)
N22—H22···O1^iii^	0.89 (3)	1.75 (3)	2.637 (9)	172 (9)
O27*A*—H27*A*···O26*A*^iv^	0.84	1.85	2.683 (9)	170
O27*B*—H27*B*···O26*B*^iv^	0.84	1.84	2.62 (6)	154
C12—H12···O11^v^	1.00	2.27	3.172 (10)	150
C23—H23···O1^iii^	0.95	2.35	3.237 (9)	156

Symmetry codes: (i) *x*−1, *y*, *z*; (ii) −*x*+1, *y*+1/2, −*z*+1/2; (iii) −*x*+1/2, −*y*+1, *z*−1/2; (iv) *x*+1/2, −*y*+1/2, −*z*; (v) *x*+1, *y*, *z*.

### (6R,7R)-7-{[(Z)-2-(2-Amino-1,3-thiazol-4-yl)-4-carboxybut-2-enoyl]amino}-8-oxo-5-thia-1-azabicyclo[4.2.0]oct-2-ene-2-carboxylic acid 2.652-hydrate (II) Crystal data

**Table:**

C_15_H_14_N_4_O_6_S_2_·2.652H_2_O	*D*_x_ = 1.582 Mg m^−^^3^
*M**_r_* = 458.21	Cu *K*α radiation, λ = 1.54178 Å
Orthorhombic, *P*2_1_2_1_2_1_	Cell parameters from 8801 reflections
*a* = 4.6690 (1) Å	θ = 3.1–77.2°
*b* = 17.8029 (4) Å	µ = 3.04 mm^−^^1^
*c* = 23.1486 (5) Å	*T* = 100 K
*V* = 1924.15 (7) Å^3^	Needle, colourless
*Z* = 4	0.1 × 0.02 × 0.02 mm
*F*(000) = 954	

### (6R,7R)-7-{[(Z)-2-(2-Amino-1,3-thiazol-4-yl)-4-carboxybut-2-enoyl]amino}-8-oxo-5-thia-1-azabicyclo[4.2.0]oct-2-ene-2-carboxylic acid 2.652-hydrate (II) Data collection

**Table:**

Bruker D8 Venture Photon-II CPAD diffractometer	4040 independent reflections
Radiation source: INCOATEC Imus micro-focus source	3605 reflections with *I* > 2σ(*I*)
Mirrors monochromator	*R*_int_ = 0.070
ω scans	θ_max_ = 78.0°, θ_min_ = 3.1°
Absorption correction: multi-scan (SADABS; Bruker, 2016)	*h* = −5→5
*T*_min_ = 0.919, *T*_max_ = 1.000	*k* = −22→21
26013 measured reflections	*l* = −28→29

### (6R,7R)-7-{[(Z)-2-(2-Amino-1,3-thiazol-4-yl)-4-carboxybut-2-enoyl]amino}-8-oxo-5-thia-1-azabicyclo[4.2.0]oct-2-ene-2-carboxylic acid 2.652-hydrate (II) Refinement

**Table:**

Refinement on *F*^2^	Hydrogen site location: mixed
Least-squares matrix: full	H atoms treated by a mixture of independent and constrained refinement
*R*[*F*^2^ > 2σ(*F*^2^)] = 0.038	*w* = 1/[σ^2^(*F*_o_^2^) + (0.0405*P*)^2^ + 0.5445*P*] where *P* = (*F*_o_^2^ + 2*F*_c_^2^)/3
*wR*(*F*^2^) = 0.085	(Δ/σ)_max_ < 0.001
*S* = 1.04	Δρ_max_ = 0.30 e Å^−^^3^
4040 reflections	Δρ_min_ = −0.23 e Å^−^^3^
317 parameters	Absolute structure: Flack *x* determined using 1302 quotients [(*I*^+^)-(*I*^-^)]/[(*I*^+^)+(*I*^-^)] (Parsons *et al.*, 2013)
7 restraints	Absolute structure parameter: 0.029 (11)

### (6R,7R)-7-{[(Z)-2-(2-Amino-1,3-thiazol-4-yl)-4-carboxybut-2-enoyl]amino}-8-oxo-5-thia-1-azabicyclo[4.2.0]oct-2-ene-2-carboxylic acid 2.652-hydrate (II) Special details

**Table:**

Geometry. All esds (except the esd in the dihedral angle between two l.s. planes) are estimated using the full covariance matrix. The cell esds are taken into account individually in the estimation of esds in distances, angles and torsion angles; correlations between esds in cell parameters are only used when they are defined by crystal symmetry. An approximate (isotropic) treatment of cell esds is used for estimating esds involving l.s. planes.

### (6R,7R)-7-{[(Z)-2-(2-Amino-1,3-thiazol-4-yl)-4-carboxybut-2-enoyl]amino}-8-oxo-5-thia-1-azabicyclo[4.2.0]oct-2-ene-2-carboxylic acid 2.652-hydrate (II) Fractional atomic coordinates and isotropic or equivalent isotropic displacement parameters (Å^2^)

**Table:**

	*x*	*y*	*z*	*U*_iso_*/*U*_eq_	Occ. (<1)
O1	0.2135 (6)	0.50647 (13)	0.50496 (10)	0.0213 (6)	
O2	0.5160 (6)	0.59068 (13)	0.54303 (10)	0.0232 (6)	
C3	0.4020 (8)	0.52690 (19)	0.54008 (14)	0.0180 (7)	
C4	0.5001 (8)	0.46858 (18)	0.58327 (14)	0.0160 (7)	
C5	0.4378 (8)	0.39600 (19)	0.57856 (14)	0.0192 (7)	
H5	0.329092	0.381498	0.545704	0.023*	
C6	0.5210 (10)	0.3343 (2)	0.61975 (15)	0.0273 (9)	
H6A	0.687457	0.307006	0.603607	0.033*	
H6B	0.360269	0.298288	0.622794	0.033*	
S7	0.6123 (2)	0.36755 (5)	0.69200 (4)	0.0228 (2)	
C8	0.8320 (8)	0.4455 (2)	0.66878 (15)	0.0186 (7)	
H8	1.025171	0.430505	0.654104	0.022*	
N9	0.6706 (6)	0.49483 (15)	0.62916 (12)	0.0163 (6)	
C10	0.6178 (8)	0.54808 (19)	0.67132 (14)	0.0167 (7)	
O11	0.4464 (5)	0.59867 (13)	0.67441 (10)	0.0198 (5)	
C12	0.8375 (8)	0.5122 (2)	0.71215 (14)	0.0172 (7)	
H12	1.024780	0.539335	0.710828	0.021*	
N13	0.7457 (7)	0.50003 (17)	0.77034 (13)	0.0172 (6)	
H13	0.572 (6)	0.491 (2)	0.7776 (16)	0.016 (10)*	
C14	0.9261 (7)	0.50079 (17)	0.81555 (14)	0.0146 (7)	
O15	1.1831 (5)	0.51428 (16)	0.81065 (11)	0.0261 (6)	
C16	0.7879 (7)	0.48572 (18)	0.87346 (14)	0.0140 (7)	
C17	0.6484 (8)	0.41288 (19)	0.87863 (14)	0.0160 (7)	
C18	0.6852 (8)	0.35256 (18)	0.84425 (15)	0.0179 (7)	
H18	0.811218	0.351654	0.812040	0.021*	
S19	0.4772 (2)	0.27702 (4)	0.86532 (4)	0.0196 (2)	
C20	0.3429 (8)	0.32934 (19)	0.92227 (15)	0.0176 (7)	
N21	0.1548 (7)	0.30362 (18)	0.95965 (14)	0.0199 (7)	
H21A	0.081 (10)	0.260 (3)	0.9539 (18)	0.029 (12)*	
H21B	0.089 (10)	0.333 (2)	0.9849 (18)	0.023 (11)*	
N22	0.4532 (6)	0.39822 (15)	0.92347 (12)	0.0149 (6)	
H22	0.398 (13)	0.430 (3)	0.949 (2)	0.056 (16)*	
C23	0.8045 (8)	0.53727 (19)	0.91527 (15)	0.0169 (7)	
H23	0.709285	0.526631	0.950597	0.020*	
C24	0.9607 (8)	0.61028 (19)	0.91095 (15)	0.0198 (7)	
H24A	0.957574	0.626679	0.870087	0.024*	
H24B	1.163328	0.601559	0.921618	0.024*	
C25	0.8476 (8)	0.6731 (2)	0.94744 (15)	0.0189 (7)	
O26	0.5947 (6)	0.66103 (15)	0.97034 (12)	0.0249 (6)	
H26	0.559 (11)	0.702 (3)	0.989 (2)	0.039 (14)*	
O27	0.9818 (6)	0.73087 (14)	0.95451 (11)	0.0270 (6)	
O31	0.9738 (6)	0.66362 (14)	0.59300 (12)	0.0247 (6)	
H31A	1.136 (7)	0.643 (3)	0.591 (2)	0.050 (16)*	
H31B	0.849 (10)	0.635 (3)	0.577 (3)	0.09 (2)*	
O32	−0.0194 (9)	0.7050 (2)	0.73219 (18)	0.0412 (14)	0.828 (10)
H32A	0.153 (9)	0.700 (5)	0.747 (3)	0.09 (3)*	0.828 (10)
H32B	−0.013 (15)	0.686 (4)	0.6976 (15)	0.07 (2)*	0.828 (10)
O33	−0.5146 (9)	0.6766 (2)	0.79978 (17)	0.0357 (14)	0.824 (12)
H33A	−0.563 (16)	0.630 (5)	0.795 (3)	0.07 (2)*	0.824 (12)
H33B	−0.382 (16)	0.689 (3)	0.774 (3)	0.04 (2)*	0.824 (12)

### (6R,7R)-7-{[(Z)-2-(2-Amino-1,3-thiazol-4-yl)-4-carboxybut-2-enoyl]amino}-8-oxo-5-thia-1-azabicyclo[4.2.0]oct-2-ene-2-carboxylic acid 2.652-hydrate (II) Atomic displacement parameters (Å^2^)

**Table:**

	*U*^11^	*U*^22^	*U*^33^	*U*^12^	*U*^13^	*U*^23^
O1	0.0269 (14)	0.0204 (12)	0.0167 (12)	−0.0034 (11)	−0.0091 (11)	0.0025 (10)
O2	0.0267 (14)	0.0194 (12)	0.0236 (12)	−0.0047 (11)	−0.0078 (12)	0.0047 (10)
C3	0.0193 (18)	0.0208 (17)	0.0138 (16)	0.0021 (14)	0.0040 (15)	−0.0003 (13)
C4	0.0169 (17)	0.0201 (16)	0.0110 (14)	0.0023 (14)	0.0027 (14)	0.0002 (12)
C5	0.0237 (19)	0.0213 (16)	0.0127 (15)	0.0040 (15)	−0.0025 (15)	−0.0010 (13)
C6	0.044 (2)	0.0194 (16)	0.0190 (17)	0.0040 (18)	−0.0067 (18)	−0.0022 (14)
S7	0.0331 (5)	0.0195 (4)	0.0158 (4)	0.0004 (4)	−0.0037 (4)	0.0026 (3)
C8	0.0152 (18)	0.0262 (18)	0.0142 (17)	0.0053 (14)	0.0007 (14)	0.0026 (14)
N9	0.0169 (15)	0.0166 (14)	0.0154 (14)	0.0009 (11)	−0.0001 (12)	0.0037 (11)
C10	0.0159 (16)	0.0187 (16)	0.0154 (16)	−0.0044 (15)	0.0031 (15)	0.0027 (13)
O11	0.0207 (13)	0.0197 (12)	0.0190 (11)	−0.0004 (10)	0.0030 (10)	0.0007 (10)
C12	0.0137 (17)	0.0257 (18)	0.0121 (15)	−0.0035 (14)	0.0020 (14)	0.0014 (13)
N13	0.0127 (14)	0.0256 (16)	0.0132 (14)	−0.0059 (13)	0.0011 (12)	0.0004 (12)
C14	0.0157 (16)	0.0144 (15)	0.0139 (15)	0.0006 (13)	−0.0001 (13)	−0.0024 (12)
O15	0.0165 (13)	0.0451 (16)	0.0166 (12)	0.0002 (11)	−0.0004 (11)	−0.0013 (12)
C16	0.0146 (16)	0.0150 (15)	0.0125 (16)	0.0024 (13)	0.0005 (13)	0.0007 (13)
C17	0.0177 (17)	0.0183 (16)	0.0122 (15)	0.0017 (14)	−0.0005 (14)	0.0006 (13)
C18	0.0236 (19)	0.0139 (16)	0.0161 (16)	−0.0004 (13)	0.0006 (15)	0.0008 (13)
S19	0.0289 (5)	0.0134 (4)	0.0165 (4)	−0.0009 (4)	0.0025 (4)	−0.0031 (3)
C20	0.0233 (18)	0.0174 (16)	0.0122 (15)	0.0005 (15)	−0.0039 (15)	−0.0001 (13)
N21	0.0277 (18)	0.0135 (15)	0.0186 (16)	−0.0040 (13)	0.0039 (14)	−0.0020 (12)
N22	0.0164 (15)	0.0145 (13)	0.0139 (13)	0.0005 (12)	0.0010 (12)	−0.0011 (11)
C23	0.0190 (19)	0.0172 (16)	0.0146 (16)	0.0012 (14)	0.0011 (14)	0.0008 (13)
C24	0.0216 (19)	0.0198 (16)	0.0180 (16)	−0.0033 (15)	0.0003 (15)	−0.0044 (13)
C25	0.0219 (19)	0.0186 (17)	0.0162 (16)	−0.0038 (15)	−0.0035 (15)	0.0014 (13)
O26	0.0243 (14)	0.0187 (12)	0.0317 (14)	−0.0015 (11)	0.0046 (12)	−0.0078 (11)
O27	0.0301 (15)	0.0207 (13)	0.0301 (14)	−0.0055 (12)	0.0043 (12)	−0.0073 (11)
O31	0.0174 (14)	0.0176 (12)	0.0390 (16)	0.0012 (12)	−0.0020 (13)	−0.0008 (11)
O32	0.038 (3)	0.039 (2)	0.046 (3)	0.0030 (19)	−0.010 (2)	−0.0041 (18)
O33	0.040 (2)	0.030 (2)	0.038 (2)	0.0079 (18)	−0.003 (2)	−0.0082 (16)

### (6R,7R)-7-{[(Z)-2-(2-Amino-1,3-thiazol-4-yl)-4-carboxybut-2-enoyl]amino}-8-oxo-5-thia-1-azabicyclo[4.2.0]oct-2-ene-2-carboxylic acid 2.652-hydrate (II) Geometric parameters (Å, º)

**Table:**

O1—C3	1.252 (4)	C17—C18	1.348 (5)
O2—C3	1.256 (4)	C17—N22	1.406 (4)
C3—C4	1.512 (5)	C18—H18	0.9500
C4—C5	1.329 (5)	C18—S19	1.729 (4)
C4—N9	1.407 (4)	S19—C20	1.732 (4)
C5—H5	0.9500	C20—N21	1.315 (5)
C5—C6	1.505 (5)	C20—N22	1.330 (4)
C6—H6A	0.9900	N21—H21A	0.86 (5)
C6—H6B	0.9900	N21—H21B	0.85 (4)
C6—S7	1.824 (4)	N22—H22	0.87 (5)
S7—C8	1.807 (4)	C23—H23	0.9500
C8—H8	1.0000	C23—C24	1.494 (5)
C8—N9	1.477 (4)	C24—H24A	0.9900
C8—C12	1.555 (5)	C24—H24B	0.9900
N9—C10	1.383 (4)	C24—C25	1.498 (5)
C10—O11	1.207 (4)	C25—O26	1.312 (5)
C10—C12	1.534 (5)	C25—O27	1.215 (4)
C12—H12	1.0000	O26—H26	0.87 (5)
C12—N13	1.430 (4)	O31—H31A	0.85 (2)
N13—H13	0.85 (2)	O31—H31B	0.86 (3)
N13—C14	1.343 (5)	O32—H32A	0.88 (3)
C14—O15	1.229 (4)	O32—H32B	0.87 (3)
C14—C16	1.512 (5)	O33—H33A	0.87 (8)
C16—C17	1.456 (5)	O33—H33B	0.88 (8)
C16—C23	1.336 (5)		
			
O1—C3—O2	126.6 (3)	O15—C14—N13	122.8 (3)
O1—C3—C4	116.3 (3)	O15—C14—C16	122.2 (3)
O2—C3—C4	117.2 (3)	C17—C16—C14	115.0 (3)
C5—C4—C3	123.2 (3)	C23—C16—C14	119.7 (3)
C5—C4—N9	120.6 (3)	C23—C16—C17	125.3 (3)
N9—C4—C3	116.2 (3)	C18—C17—C16	127.2 (3)
C4—C5—H5	116.5	C18—C17—N22	111.8 (3)
C4—C5—C6	126.9 (3)	N22—C17—C16	121.1 (3)
C6—C5—H5	116.5	C17—C18—H18	123.8
C5—C6—H6A	108.8	C17—C18—S19	112.4 (3)
C5—C6—H6B	108.8	S19—C18—H18	123.8
C5—C6—S7	113.9 (2)	C18—S19—C20	89.99 (17)
H6A—C6—H6B	107.7	N21—C20—S19	123.7 (3)
S7—C6—H6A	108.8	N21—C20—N22	124.5 (3)
S7—C6—H6B	108.8	N22—C20—S19	111.8 (3)
C8—S7—C6	96.25 (17)	C20—N21—H21A	119 (3)
S7—C8—H8	114.1	C20—N21—H21B	119 (3)
N9—C8—S7	110.6 (2)	H21A—N21—H21B	122 (4)
N9—C8—H8	114.1	C17—N22—H22	126 (4)
N9—C8—C12	87.4 (2)	C20—N22—C17	114.0 (3)
C12—C8—S7	113.8 (2)	C20—N22—H22	120 (4)
C12—C8—H8	114.1	C16—C23—H23	117.4
C4—N9—C8	124.0 (3)	C16—C23—C24	125.3 (3)
C10—N9—C4	131.3 (3)	C24—C23—H23	117.4
C10—N9—C8	93.5 (3)	C23—C24—H24A	108.3
N9—C10—C12	91.7 (3)	C23—C24—H24B	108.3
O11—C10—N9	132.2 (3)	C23—C24—C25	116.1 (3)
O11—C10—C12	135.9 (3)	H24A—C24—H24B	107.4
C8—C12—H12	111.3	C25—C24—H24A	108.3
C10—C12—C8	84.8 (2)	C25—C24—H24B	108.3
C10—C12—H12	111.3	O26—C25—C24	115.0 (3)
N13—C12—C8	119.2 (3)	O27—C25—C24	121.7 (3)
N13—C12—C10	116.3 (3)	O27—C25—O26	123.3 (3)
N13—C12—H12	111.3	C25—O26—H26	104 (3)
C12—N13—H13	120 (3)	H31A—O31—H31B	109 (4)
C14—N13—C12	123.0 (3)	H32A—O32—H32B	107 (5)
C14—N13—H13	117 (3)	H33A—O33—H33B	110 (6)
N13—C14—C16	114.9 (3)		
			
O1—C3—C4—C5	12.9 (5)	O11—C10—C12—C8	162.7 (4)
O1—C3—C4—N9	−168.6 (3)	O11—C10—C12—N13	42.6 (6)
O2—C3—C4—C5	−167.3 (4)	C12—C8—N9—C4	−159.5 (3)
O2—C3—C4—N9	11.2 (5)	C12—C8—N9—C10	−12.5 (3)
C3—C4—C5—C6	−178.8 (4)	C12—N13—C14—O15	2.3 (5)
C3—C4—N9—C8	−167.6 (3)	C12—N13—C14—C16	−179.2 (3)
C3—C4—N9—C10	58.7 (5)	N13—C14—C16—C17	60.6 (4)
C4—C5—C6—S7	20.6 (5)	N13—C14—C16—C23	−120.4 (4)
C4—N9—C10—O11	−19.3 (6)	C14—C16—C17—C18	16.9 (5)
C4—N9—C10—C12	155.7 (3)	C14—C16—C17—N22	−164.0 (3)
C5—C4—N9—C8	10.9 (5)	C14—C16—C23—C24	−2.2 (5)
C5—C4—N9—C10	−122.7 (4)	O15—C14—C16—C17	−120.9 (4)
C5—C6—S7—C8	−44.7 (3)	O15—C14—C16—C23	58.1 (5)
C6—S7—C8—N9	55.0 (3)	C16—C17—C18—S19	−179.8 (3)
C6—S7—C8—C12	151.5 (3)	C16—C17—N22—C20	179.7 (3)
S7—C8—N9—C4	−45.0 (4)	C16—C23—C24—C25	151.9 (3)
S7—C8—N9—C10	102.0 (3)	C17—C16—C23—C24	176.6 (3)
S7—C8—C12—C10	−100.1 (3)	C17—C18—S19—C20	−0.6 (3)
S7—C8—C12—N13	17.2 (4)	C18—C17—N22—C20	−1.0 (4)
C8—N9—C10—O11	−162.4 (4)	C18—S19—C20—N21	−179.9 (3)
C8—N9—C10—C12	12.7 (3)	C18—S19—C20—N22	0.0 (3)
C8—C12—N13—C14	110.4 (4)	S19—C20—N22—C17	0.6 (4)
N9—C4—C5—C6	2.7 (6)	N21—C20—N22—C17	−179.5 (3)
N9—C8—C12—C10	11.3 (2)	N22—C17—C18—S19	1.0 (4)
N9—C8—C12—N13	128.6 (3)	C23—C16—C17—C18	−162.1 (4)
N9—C10—C12—C8	−12.0 (2)	C23—C16—C17—N22	17.0 (5)
N9—C10—C12—N13	−132.2 (3)	C23—C24—C25—O26	−12.3 (5)
C10—C12—N13—C14	−150.3 (3)	C23—C24—C25—O27	168.2 (3)

### (6R,7R)-7-{[(Z)-2-(2-Amino-1,3-thiazol-4-yl)-4-carboxybut-2-enoyl]amino}-8-oxo-5-thia-1-azabicyclo[4.2.0]oct-2-ene-2-carboxylic acid 2.652-hydrate (II) Hydrogen-bond geometry (Å, º)

**Table:**

*D*—H···*A*	*D*—H	H···*A*	*D*···*A*	*D*—H···*A*
N13—H13···O15^i^	0.85 (2)	2.01 (3)	2.799 (4)	154 (4)
N21—H21*A*···O31^ii^	0.86 (5)	2.05 (5)	2.838 (4)	153 (4)
N21—H21*B*···O2^iii^	0.85 (4)	1.97 (5)	2.811 (4)	173 (4)
N22—H22···O1^iii^	0.87 (5)	1.78 (5)	2.654 (4)	178 (5)
O26—H26···O27^iv^	0.87 (5)	1.80 (5)	2.647 (4)	164 (4)
O31—H31*A*···O2^v^	0.85 (2)	2.29 (3)	3.071 (4)	154 (5)
O31—H31*B*···O2	0.86 (3)	1.91 (3)	2.756 (4)	167 (6)
O32—H32*A*···O33^v^	0.88 (3)	2.02 (3)	2.874 (6)	164 (8)
O32—H32*B*···O31^i^	0.87 (3)	2.46 (3)	3.305 (5)	167 (6)
O33—H33*A*···O15^vi^	0.87 (8)	2.40 (8)	3.226 (5)	159 (6)
O33—H33*B*···O32	0.88 (8)	1.97 (8)	2.837 (6)	165 (6)
C12—H12···O11^v^	1.00	2.39	3.349 (4)	161
C23—H23···O1^iii^	0.95	2.41	3.281 (4)	152
C24—H24*B*···O26^v^	0.99	2.54	3.387 (5)	143

Symmetry codes: (i) *x*−1, *y*, *z*; (ii) −*x*+1, *y*−1/2, −*z*+3/2; (iii) −*x*+1/2, −*y*+1, *z*+1/2; (iv) *x*−1/2, −*y*+3/2, −*z*+2; (v) *x*+1, *y*, *z*; (vi) *x*−2, *y*, *z*.
